# Differential expression patterns of two delta‐9‐acyl‐CoA desaturases in *Thitarodes pui* (Lepidoptera: Hepialidae) during different seasons and cold exposure

**DOI:** 10.1002/ece3.2792

**Published:** 2017-02-19

**Authors:** Qiang Min, Shiyu Cheng, Jianfei Xi, Tianrong Xin, Bin Xia, Zhiwen Zou

**Affiliations:** ^1^ School of life sciencesNanchang UniversityNanchangChina

**Keywords:** acclimation, delta‐9‐acyl‐CoA desaturases, experimental evolution, thermal adaptation, *Thitarodes pui*

## Abstract

*Thitarodes pui* larvae have a limited distribution in the Tibetan Plateau and are the host of a parasitic fungus, *Ophiocordyceps sinensis*. Low temperature is a main environmental stress. However, understanding of *T. pui* cold adaptation mechanisms is insufficient. Delta‐9‐acyl‐CoA desaturase (D9D) is closely correlated with cold adaptation for many organisms. To further understand the cold adaptation processes in *T. pui* larvae, two *D9D*s, *TpdesatA* and *TpdesatB* were sequenced, and expression patterns were investigated during different seasons and cold exposure (under 0°C) in the laboratory. The full lengths of two cDNAs are 1,290 bp and 1,603 bp, and the ORFs encode a polypeptide of 348 and 359 amino acids, respectively. Four transmembrane domains, three conserved histidine residues and five hydrophobic regions exist in these two sequences. The expression level of *TpdesatA* is up‐regulated in the long‐term cold exposure and negatively correlated with temperature in seasonal patterns. *TpdesatB* responds to cold temperature in short‐term cold exposure and positively corresponds temporarily in seasonal expression. Two *D9D*s may have different substrate specificities, *TpdesatA* tends to use C16:0 and C18:0 as substrate while *TpdesatB* prefers C18:0. In conclusion, *TpdesatA* may play a very important role in *T. pui* cold tolerance and *TpdesatB* regulates function in short‐term cold exposure and content change of fatty acids in the body.

## 1 Introduction

For overwintering insects, low temperature is one of the most serious environmental stresses affecting their survival. In fact, overwintering insects survive low temperature due to a variety of physiological and biochemical adaptations (Baust & Rojas, 1985; Clark et al., 2009). Until recently, cold hardiness in insects was most often discussed in terms of cryoprotectants, membrane lipids, and heat‐shock proteins (Storey & Storey, 2012; Teets & Denlinger, 2013; Yocum, 2001). The transition of cell membrane lipids from a liquid crystalline phase to a gel phase is an important cause of cold injuries under nonfreezing conditions (Michaud & Denlinger, 2006). Further investigations revealed that accumulation of unsaturated fatty acids (UFAs) contributed to the fluidity of cellar membranes which are susceptible to cold **(**Khani, Moharamipour, & Barzegar, 2007; Koštá, Berkova, & Šimek, 2003; Michaud & Denlinger, 2006). In most cases, more UFAs and less saturated fatty acids (SFAs) were detected in response to cold exposure (Los & Murata, 2004; Yi, Guo, Zou, & Zhang, 2015). Studies on the composition of cell membrane lipids in many species of microorganisms, plants, and animals under different temperatures have revealed the universal occurrence of remodeling of cell membrane lipids in response to changes in ambient temperature, a phenomenon known as homeoviscous adaptation (Hazel, 1995; Teets & Denlinger, 2013). The increasing UFAs are considered to play a role in maintaining the liquid crystalline phase at low temperatures (Kayukawa, Chen, Hoshizaki, & Ishikawa, 2007).

Delta‐9‐acyl‐CoA desaturase (D9D) is an important enzyme that introduces a double bond into SFAs, and has been shown to play an essential role in cold hardiness by increasing the ratio of UFAs to SFAs in cell membranes (Hsieh & Kuo, 2005; Tiku, Gracey, Macartney, Beynon, & Cossins, 1996). Certain groups of reports demonstrated that up‐regulation of the *D9D* gene occurs during cold exposure. Tiku et al. (1996) indicated that transcription of the *D9D* gene increased tenfold in the liver of cold‐exposed carp. Similar findings were also proved in *Oreochromis niloticus* (Zerai, Fitzsimmons, & Collier, 2010), as well as *Chanos chanos* and *Ctenopharyngodon idella* (Hsieh & Kuo, 2005). For insects, the *D9D* gene was firstly proved to participate in cold adaptation mechanisms in *Delia antique*. In that study, twofold to tenfold up‐regulation of the *D9D* gene was induced in brain tissues, malpighian tubules, and the midgut when *D. antique* was exposed to cold (Kayukawa et al., 2007). This same result occurred in *Sarcophaga crassipalpis* (Rinehart, Robich, & Denlinger, 2010), *Folsomia candida* (Waagner, Holmstrup, Bayley, & Jesper, 2013), and *Aedes albopictus* (Reynolds et al. 2012). Meanwhile, another kind of D9D, which was associated with dietary alterations, was found in *Cyprinus carpio* and *Acheta domesticus* (Batcabe, Howell, Blomquist, & Borgeson, 2000; Polley et al., 2003).

**Figure 1:**
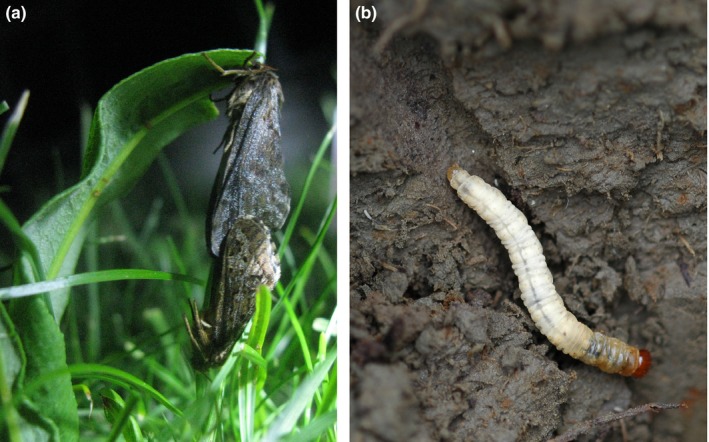
Adults (a) and larva (b) of *Thitarodes pui*

*Thitarodes pui* (Zhang et al.) (Lepidoptera: Hepialidae) (Figure 1) was first reported as *Hepialus pui* (Zhang, Gu, & Liu, 2007) in China but was later moved to the genus *Thitarodes* (Zou, 2009; Zou, Liu, & Zhang, 2010). Larvae of *Thitarodes* in Southeast Asia are the host of the fungus *Ophiocordyceps sinensis* (Berkeley) Saccardo (Dong Chong Xia Cao in Chinese) (Winkler, 2009), which is one of the most valuable resources for traditional Chinese medicine (Buenz, Bauer, Osmundson, & Motley, 2005; Yue, Ye, Lin, & Zhou, 2013; Zhu, Halpern, & Jones, 1998). *T. pui* has a limited distribution of 4,100–5,000 m surrounding Mount Segrila in Tibet (Zhang et al., 2007). In this region, the average annual temperature is below 5°C and the soil is periodically frozen and thawed, and low temperature is considered a main environmental stress for *T. pui* (Yi, Guo, et al., 2015). Nevertheless, *Thitarodes* larvae can endure extreme temperatures at −12 and −20°C, but the mechanism for this cold tolerance is unclear (Yang, Li, Shu, & Yang, 1996).

Our previous work indicated that proteins, total sugar, and total fat in the hemolymph of *T. pui* larvae showed negative correlation with soil temperature (Yi, Zhang, Guo, Min, & Zou, 2015). In addition, *HSP90* of *T. pui*, rather than *HSP70*, responds to temperature changes and potentially plays a key role in cold tolerance (Zou, Sun, Li, & Zhang, 2011). In addition, trehalose‐6‐phosphate synthase is involved in the complicated cold adaptation process in *T*. *pui* (Min et al., 2016). To obtain a further understanding of cold adaptation, two *D9D* genes (*TpdesatA* and *TpdesatB*) were sequenced in *T. pui* larvae and their expression patterns were investigated by real‐time PCR during different seasons and cold exposure under 0°C in the laboratory. The results might serve to build a framework for comprehensively understanding the biology and molecular mechanisms of *T. pui* adaptation to thermal stress.

## 2 Materials and methods

### 2.1 Temperature measurement of soil with *T. pui* larvae

Temperature of soil at 20 cm below the surface was measured with Hobo Pro temperature and RH data logger (Model H08‐032‐08, Eco‐tech Co. LTD, USA). The data logger was set to record the temperature every 30 min, and the data were downloaded every 30 days with BoxCar Pro software (version 4.3, Onset Computer Corporation, USA) (Zou et al., 2011).

### 2.2 Insect collection and cold exposure regime

The investigation of seasonal expression patterns was processed from July 2008 to June 2009. In the middle of every month, six individuals of the sixth instar *T. pui* larvae were collected from Mt. Segrila (4,156 m, 29°37′N, 94°37′E) in the Tibetan Plateau, and these samples were used to transcription level analysis. Experiments under cold exposure were processed from July to August in 2013. More than 100 individuals of sixth instar *T. pui* larvae were collected in July 2013 at the same area, then fed in soil at laboratory, and the environment‐controlled at 10°C; after fifteen days, they were used to cold exposure experience. Ten individuals were used as a control group. The other were reared at 0°C (Thermo Scientific Precision, USA) and collected at different times, including short term (1, 3, 6, 12 hr), midterm (24, 48, 72 hr, 5 days), and long term (7, 10, and 15 days).

### 2.3 Collection of fat body

All samples were dissected to obtain fat bodies. The fat bodies isolated from two larvae were mixed and then stored in RNA protect solution (TaKaRa, Japan) at −80°C.

### 2.4 Cloning the full‐length cDNA of two *D9D* genes

Total RNA was extracted from the fat body of one individual using Trizol Reagent Kit (Invitrogen, USA) according to the manufacturer's instructions, then dissolved by 30 μl diethylpyrocarbonate (DEPC) water and stored at −80°C. The RNA was quantitated by NanoDrop 2000 (BioSpec‐mini, Shimadzu) and transferred to cDNA by using AMV reverse transcriptase (TaKaRa, Dalian, China) under the manufacturer's protocol. Degenerate primers were designed based on the conserved amino acid sequences of known *D9D* genes of other Lepidoptera insects (Table 1) and then used to amplify the initial segments of two *D9D* genes. The PCR was conducted with 30 cycles under condition of 30 s at 94°C for denaturation, 30 s at 45°C for annealing, 1 min at 72°C for extension. Specific primers for 5′‐RACE (Rapid amplification of cDNA ends) and 3′‐RACE (Table 1) were synthesized based on the initial segments of two *D9D* genes. The 5′ and 3′ RACE were processed using the SMART RACE cDNA Amplification Kit (Clontech, CA, USA). The PCR was placed in 50 μl volume with 30 cycles under the condition of 30 s at 94°C, 30 s at 55°C, 2 min at 72°C.

**Table 1:** Primers used for cloning and expression analysis of two D9D in *Thitarodes pui*

Fragment	Primer	Primer sequence (F/R) 5′→ 3′
Tpdesat	desF1	TGGGCDCACAARWSHTAYAA
	desF2	GAYCAYMGNATGCAYCAYAA
	desF3	GAYGCBGAYCCNCAYAAYGC
	desR1	TGRTAGTTGTGGAADCCYTC
	desR2	TTVADRTCRTAWGCCCA
TpdesatA	TdAF1	TTTTCTCTCATATGGGCTGGC
	TdAF2	TCCGTCAGCCTGCTTACCCT
	TdAR1	GGCTACGAAAAACGCAG
	TdAR2	CTTCTGAAATGTAACGATGGGGT
	TdAR3	ATAAGCCAGCCCATATGAGAGAA
	QdesatAF	CGTCAGCCTGCTTACCCTTG
	QdesatAR	GCCCGTTCGTATGATCCTCTTC
TpdesatB	TdBF1	TGATACAGACGCCGACCCG
	TdBF2	TGCCGCTTGTCTGCTTCATT
	TdBF3	CGACCCTATCCTAGCCTTCCA
	TdBR1	GGATAGGGTCGTTCTCG
	TdBR2	TGTGCGGGTCGGCGTCTGTATC
	TdBR3	CTATCACAGAGTCCTGGAATGC
	QdesatBF	TGATACAGACGCCGACCCG
	QdesatBR	GGCAAAATGAAGCAGACAAGC
β‐Actin	QActinF	TAACCCCAAAGCGAACAGAGA
	QActinR	GCCAAGTCCAGACGGAGAATG

### 2.5 Sequence analysis

The obtained fragments of two *D9D* genes were assembled by DNASTAR and the ORFs were identified through ORF Finder (Thompson, Higgins, & Gibson, 1994), respectively. Amino acid sequences were deduced from the corresponding cDNA sequences by using the translation tool on the ExPASy Proteomics Web site, and analogs were searched by BLASTP at the NCBI (Zou et al., 2011). The molecular weight (MW) and isoelectric point (PI) of the deduced amino acid sequences were predicted from the ExPASy Proteomics Web site. Analysis of the transmembrane domains and hydrophobic regions were performed using Kyte–Doolittle hydropathy plots in DNASTAR. Multiple alignments were performed among *TpdesatA*,* TpdesatB* as well as the analogous amino acid sequences by DNAMAN. Finally, base on the amino acid sequences of known *D9D* genes in Lepidoptera and Dipteran from GenBank (Kayukawa et al., 2007), phylogenetic tree was constructed using the neighbor‐joining method in MEGA software with 1,000 bootstrap replications.

### 2.6 Quantitative analysis of two *D9D* genes

The seasonal expression and cold adaptation changes of two *D9D* genes were investigated through RT‐PCR in CFX96™ Real‐Time System. Two pairs of primers were designed for the quantitative analysis of two genes as well as a pair of primers for the control (β‐actin) (Table 1). The reaction was performed following the manufacturer's instructions of SYBR^®^ Premix Ex Taq™ (TaKaRa, Dalian, China) with the conditions as followed: 3 min at 95°C followed by 40 cycles of 95°C for 15 s, 30 s at 60°C and 30 s at 72°C. The remaining curve analysis at the end of program was used to test the specificity of primers. Experimental operation was repeated three times for each group. 2^−ΔΔCt^ method was used to determine the expression profiles of *TpdesatA* and *TpdesatB*. The relative mRNA levels of *TpdesatA* and *TpdesatB* in July and 0 hr were set as 1, respectively.

### 2.7 Statistical analysis

Means and variances of treatments were analyzed using SPSS program (version 19.0, IBM Inc., USA), and the relative mRNA levels of *D9D* in July or control group was set as 1. All data were shown as mean ± *SD*. The means were compared with variance (ANOVA) and Tukey's studentized range test with the level of significant difference at *p *<* *.05 and highly significant difference at *p *<* *.01.

## 3 Results

### 3.1 Sequence identification and characterization of two *D9D* genes

**Figure 2:**
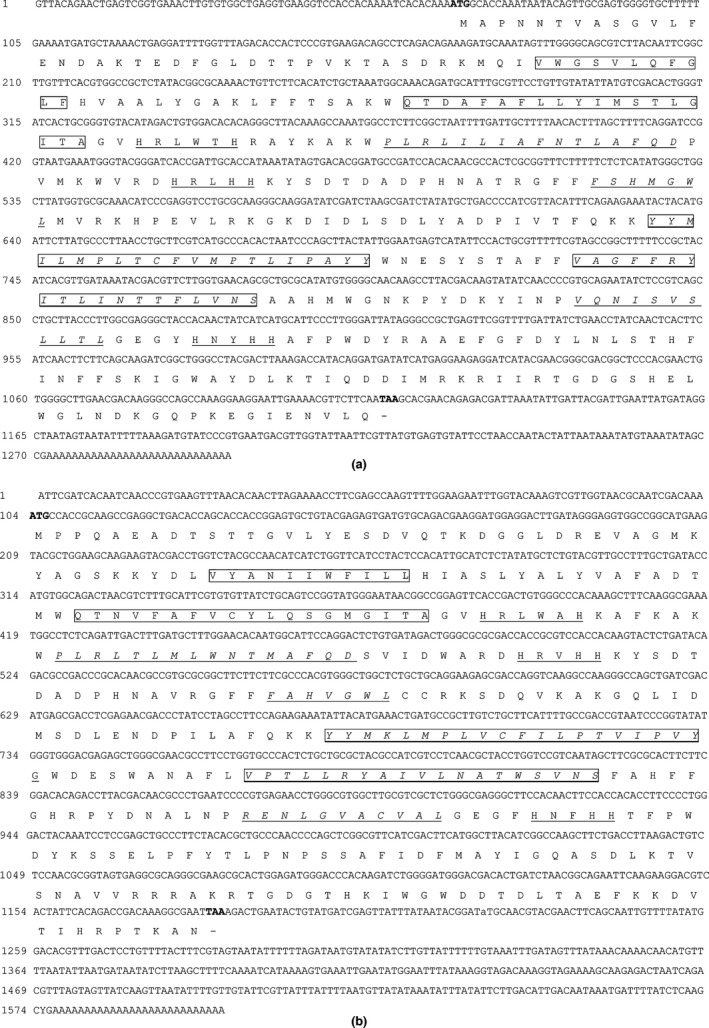
Nucleotide and deduced amino acid sequences of *TpdesatA* (a) and *TpdesatB* (b) The start and stop codons were showed as bold. Four transmembrane domains and three conserved histidine residues were boxed and underlined, separately. Five hydrophobic regions were leaned and underlined

The full length of two *D9D* genes, *TpdesatA* and *TpdesatB*, were obtained through overlapping PCR and RACE. They are 1,290 bp and 1,603 bp, and their nucleotide sequences and deduced amino acid sequences are shown in Figure 2a,b, respectively. Their nucleotide sequences have been deposited in NCBI GenBank with accession numbers GU126468 and GU205814, respectively.

**Figure 3:**
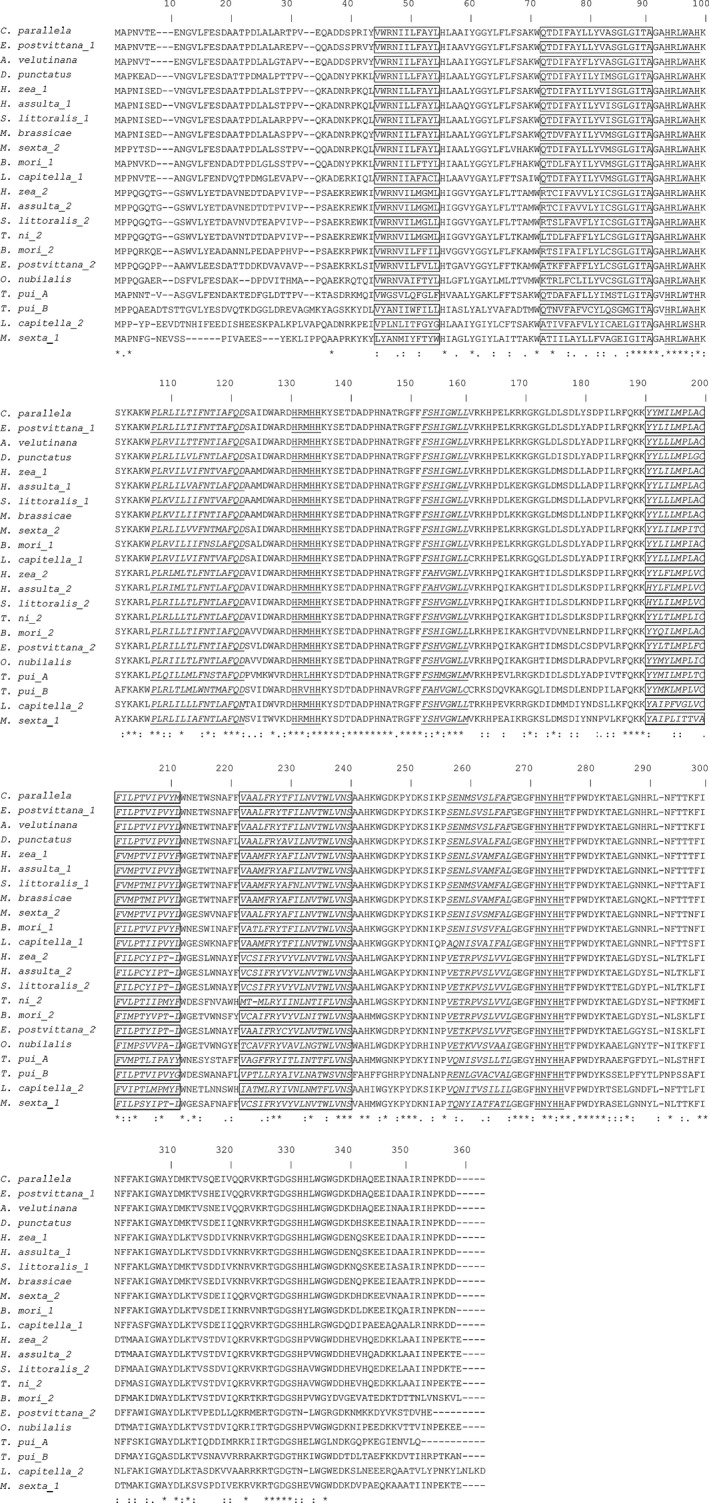
Multiple alignment of *Tpdesat* amino acids in insects Four transmembrane domains and three conserved histidine residues were boxed and underlined, separately. Five hydrophobic regions were leaned and underlined

The full length of *TpdesatA* cDNA contains 65 bp in the 5′‐untranslated region (UTR), 1,041 bp in the open reading frame (ORF) and 184 bp in 3′‐UTR. The ORF encodes a polypeptide of 348 amino acids. The inferred molecular mass of the mature protein is 107.5 kDa with an estimated PI of 5.02 (Figure 2a). The *TpdesatB* includes 103 bp in 5′‐UTR, 420 bp in 3′‐UTR and 1,080 bp in an ORF encoding a polypeptide of 359 amino acids with MWs of 133.4 kDa and PI of 4.96 (Figure 2b). Otherwise, three histidine clusters can be found in two D9D (HXXXH, HXXHH, and EXXHXXHH) (Figure 2a,b). The presence of five hydrophobic regions and four transmembrane domains in *TpdesatA* and *TpdesatB* was revealed with Kyte–Doolittle hydropathy analysis. The amino acids of *TpdesatA* and *TpdesatB* were aligned to those *D9D* genes of other 12 species, and analysis results showed that *TpdesatA* is similar to *Manduca sexta* (65.6%), *Bombyx mori* (65.6%), *Lampronia capitella* (65.3%); *TpdesatB* showed a similarity with *L. capitella* (59.4%) and *Dendrolimus punctatus* (59.4%), followed by *Epiphyas postvittana* (59.1%). Otherwise, the similarity of *TpdesatA* and *TpdesatB* is 45.5%. Same feature sequences were also existed in others species D9D sequence (Figure 3).

**Figure 4:**
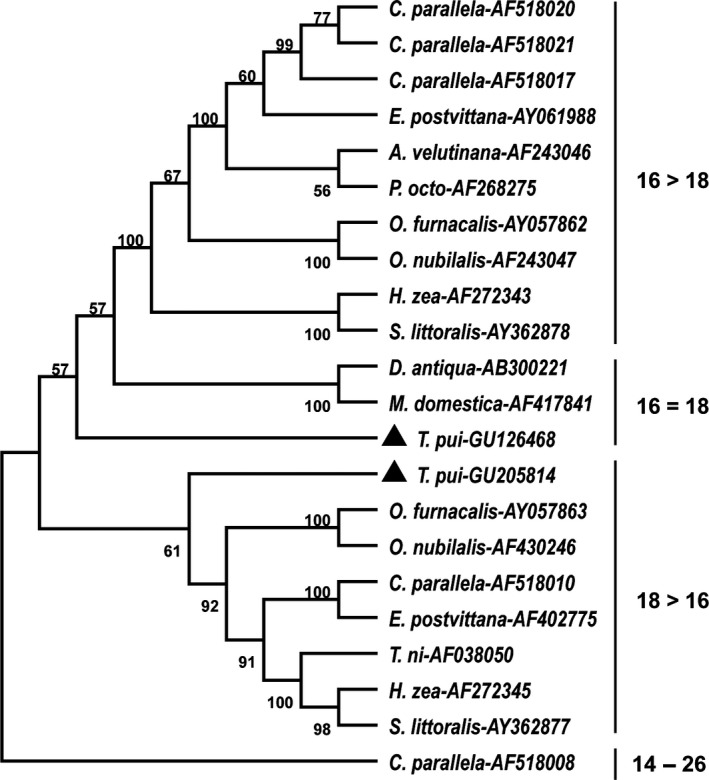
A neighbor‐joining tree of delta‐9‐acyl‐CoA desaturases in known lepidopteran and dipteran insects Bootstrap probabilities with 1,000 replicates, Genbank sequence accession numbers are given after the abbreviated species name (*A. velutinana*,* Argyrotaenia velutinana*;* C. parallela*,* Choristoneura parallela*;* D. antique*,* Delia antique*;* E. postvittana*,* Epiphyas postvittana*;* H. zea*,* Helicoverpa zea*;* M. domestica*,* Musca domestica*;* O. furnacalis*,* O. furnacalis*;* O. nubilalis*,* Ostrinia nubilalis*;* P. octo*,* Planotortrix octo*;* S. littoralis*,* Spodoptera littoralis*;* T. ni*,* Trichoplusia ni*)

A phylogenetic tree was constructed by the neighbor‐joining method, based on amino acid sequences of 20 known *D9D* in Lepidopteran and Dipteran. Four denatures groups with different substrate specificities were clearly identified as follows (substrate preferences are indicated in parentheses): △9 (16 > 18), △9 (16 = 18), △9 (18 > 16) and △9 (14–26), respectively. It was shown that *TpdesatA* gene belongs to the △9 (16 = 18) group and *TpdesatB* gene belongs to the △9 (18 > 16) (Figure 4).

### 3.2 Seasonal expression patterns of two *D9D* genes

**Figure 5:**
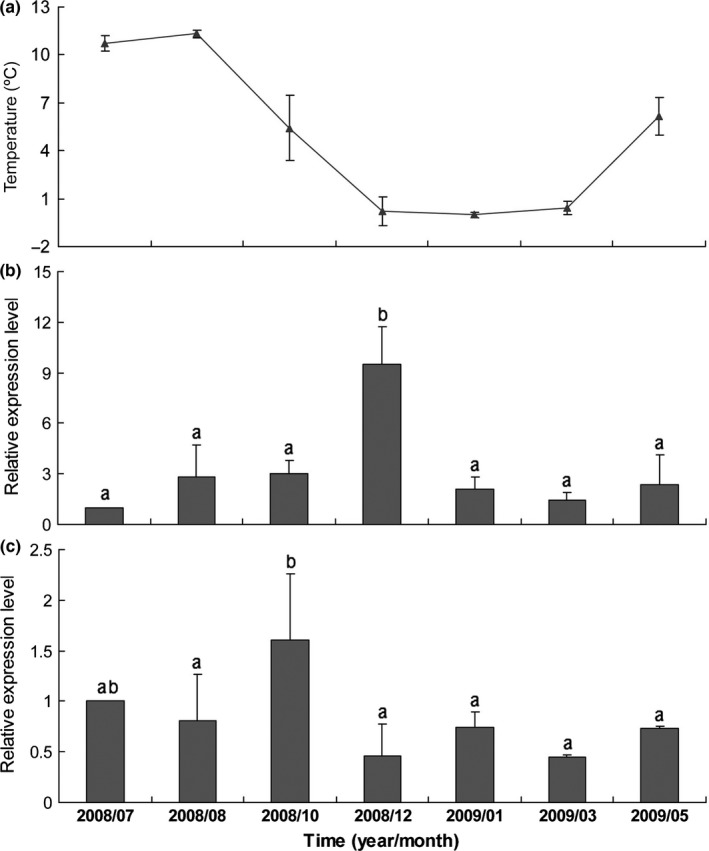
Relative mRNA levels of *TpdesatA* (b) and *TpdesatB* (c) of *Thitarodes pui* in different seasons with different soil temperature (a). Data represent means ± *SD* from three replicate experiments (*p *<* *.05), actin gene was used as reference one

Quantitative analysis was performed to indicate the seasonal expression patterns of two *D9D* genes through RT‐PCR. The soil temperature kept low level in whole year (Figure 5a). As shown in Figure 5b, the expression of *TpdesatA* exhibited a negative correlation with temperature (*y *=* *4.286 − 0.227*x*) (r = −.388, *p *=* *.390). The transcription of *TpdesatA* reached the highest level in December while the temperature remained at low level. In spite of the lowest level in March and July, the expression of *TpdesatA* remained stable in January, May, August as well as October. Expression of *TpdesatB* showed a positive correlation with soil temperature (*y *=* *0.656 + 0.035*x*) (r* *=* *.437, *p *=* *.326). During the investigation, the expression of *TpdesatB* sustained in high level in July, August, and October while it dropped and remained at low level from December to May (Figure 5c).

### 3.3 Expression patterns of two *D9D* genes during cold exposure under 0°C

**Figure 6:**
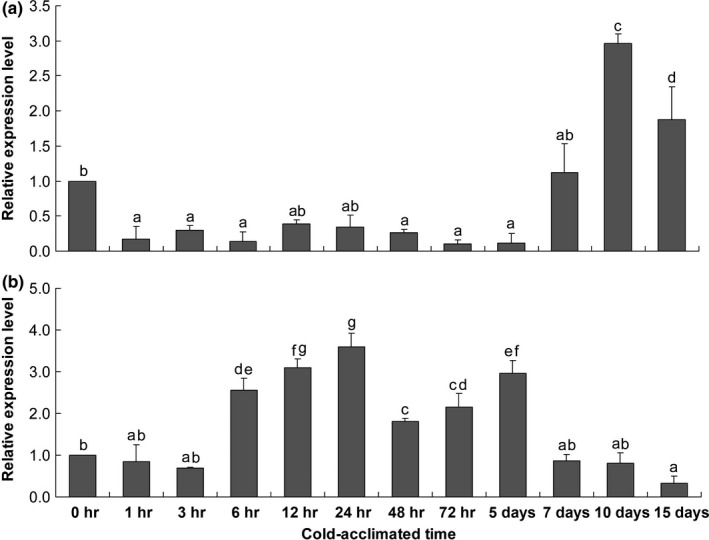
Relative mRNA levels of *TpdesatA* (a) and *TpdesatB* (b) of *Thitarodes pui* under different cold exposure at 0°C Data represent means ± *SD* from three replicate experiments. Different letters indicate significant differences (*p *<* *.05), actin gene was used as the reference one

0°C was set to explore the cold adaptation mechanism under stable cold exposure in laboratory. Significant change was detected in the expression level of *TpdesatA* during the cold stress (*F*_11, 35_ = 40.777, *p *<* *.001). In the short‐term and midterm cold exposure, *TpdesatA* was stable, and remained at a low level from 1 hr to 5 days with no substantial change detected (Figure 6a). In the long‐term cold exposure, the expression of *TpdesatA* increased from 5 days to the highest level at 10 days (2.43‐fold) and slightly declined to 1.88‐fold at 15 days. Expression of *TpdesatB* was significantly affected by cold exposure (*F*_11, 35_ = 109.469, *p *<* *.001), the expression of *TpdesatB* was up‐regulated from 6 hr (2.55‐fold) to 5 days (2.97‐fold) with a highest level appeared at 24 hr (3.59‐fold), and *TpdesatB* was down‐regulated before 3 hr and after 7 days.

## 4 Discussion

As a plateau insect with high cold tolerance, a series of physiological and biochemical mechanisms are evolved in *T. pui*. The proteins, total sugar, and total fat in the hemolymph as well as the fatty acid in whole body had the negative correlation with soil temperature (Yi, Guo, et al., 2015; Yi, Zhang, et al., 2015). Moreover, trehalose‐6‐phosphate synthase, *HSP90* of *T. pui*, rather than *HSP70*, responds to temperature changes (Min et al., 2016; Zou et al., 2011). In this paper, *TpdesatA* and *TpdesatB* were found to have the relation with cold tolerance of *T. pui*.

In *T. pui* larvae, the lipid content changes in response to soil temperature. In phospholipids, C18:1 and C18:2, showed significant negative correlation with soil temperature. However, the fluctuation soil temperature did not cause any significant changes in any of the individual's triacylglycerols fatty acids (Yi, Guo, et al., 2015), to which the molecular mechanism remains unknown. D9D plays an essential role in cold hardiness, by increasing the ratio of unsaturated and saturated fatty acids (UFA/SFA) (Rinehart et al., 2010; Tiku et al., 1996). In this study, two *D9D* genes were isolated for the first time in *Thitarodes* insects, and their expression patterns were investigated during different seasons and cold exposure under 0°C.

The two *D9D* genes separately encoded 346 AA and 359 AA amino acids, which correspond to the size range in other insects from NCBI. Alignment of two *D9D* genes and isoforms of thirteen other insects revealed *D9D* genes exist in several highly conserved regions. Four transmembrane domains existed in D9D, suggesting that the sequence spans the lipid bilayer of membranes four times (Kayukawa et al., 2007; Los & Murata, 1998). Three histidine residues in these *D9D* genes were the highly conserved regions which are catalytically essential in desaturases (Shanklin, Whittle, & Fox, 1994). These histidine residues are suggested to combine with iron atoms at the catalytic center (Los & Murata, 1998). According to the N‐J tree that was constructed in current study, two *D9D* genes in *T. pui* occurred in two independent clades, indicating that two *D9D* has different substrate specificities.

D9D desaturase is a key enzyme in synthetic pathway of UFAs, contributing to the formation of C16:1, C18:1, and C18:2. And these three UFAs are crucial to sustaining the fluidity of membranes under cold conditions (Kayukawa et al., 2007; Khani et al., 2007; Miyazaki, Kayukawa, Chen, Nomura, & Ishikawa, 2006). Previous investigations had proved that *D9D* gene was critical for cold adaptation in fish (Tiku et al., 1996), bacteria (Sakamoto & Bryant, 1997), and plants (Vega, Del Rio, Bamberg, & Palta, 2004). In this study, the expression level of *TpdesatA* was up‐regulated in the long‐term cold exposure and remained at a low level at short term and midterm; this indicates that *TpdesatA* contributes to long‐term cold hardiness.

It was shown that seasonal expression patterns of *TpdesatA* exhibited a negative correlation with soil temperature (r = −.388, *p *=* *.390). Kayukawa et al. (2007) proved that the expression of *D9D* increased to enhance the cold hardiness in *Delia antiqua* through up‐regulating the abundance of C16:1 and C18:1. So far, the same results were seen in *S. crassipalpis* (Rinehart et al., 2010), *A. albopictus* (Reynolds, Poelchau, Rahman, Armbruster, & Denlinger, 2012) and *F. candida* (Waagner et al., 2013). At the same time, seasonal cold‐hardening is defined as cold‐hardening that requires at least days to weeks for induction (Teets & Denlinger, 2013). Therefore, we suggest that *TpdesatA* has contributed to seasonal cold‐hardening. In phospholipids of *T. pui* larvae, C18:1 was the most abundant UFAs and exhibited a weak negative correlation with soil temperature. C18:2, the second abundant UFA, was highly accumulated at early days of overwintering and fluctuated in lower levels during warmer seasons. Prewinter accumulation was also detected in C18:3 (Yi, Guo, et al., 2015). These three UFAs content change in phospholipids may associate with the regulation of *TpdesatA*. Moreover, C16:0 was the second abundant in triacylglycerols, with a significant negative correlation with soil temperature (Yi, Guo, et al., 2015). At the same time, *TpdesatA* gene was clustered in the △9 (16 = 18) group in phylogenetic tree. It indicates that *TpdesatA* works in seasonal cold‐hardening, and C16:0 and C18:0 served as main substrate in triacylglycerols and phospholipids, respectively.

During cold exposure at 0°C, *TpdesatB* up‐regulated from 6 hr to 5 days and down‐regulate after 5 days; this indicates that *TpdesatB* may responds to cold temperature in short‐term cold exposure. The same results were obtained in the winter diapause pupae of *D. antiqua* (Hao et al., 2012). In the seasonal expression pattern of *TpdesatB*, it remained at high levels in summer and dropped in winter. It is obvious that high temperature contributed to the transcription of *TpdesatB* and low temperature suppressed the process (r* *=* *.437, *p *=* *.326). Polley et al. (2003) indicated that one *D9D* gene (Cds 1) in carp was well expressed at 30°C and repressed at 15°C, and the regulated pattern was associated with dietary. Down‐regulation in two *D9D*s was induced in *Drosophila montana* (*desat 1*) and *Drosophila virilis* (*desat 2*) by cold acclimatization (Vesala, Salminen, Laiho, Hoikkala, & Kankare, 2012), and there is also evidence that desaturases function in stress resistance (Greenberg, Moran, Coyne, & Wu, 2003). Based on the results showed above, *TpdesatB* seems to be not responsible for seasonal cold‐hardening. Many researchers believed that the UFAs accompanied by triacylglycerols increased and functioned as energy resources to help organism overcome cold (Joanisse & Storey, 1996; Teets & Denlinger, 2013). Therefore, *TpdesatB* may act as important short‐term regulate substance in cold exposure and caused the change of the proportion of fatty acids in the body. Meanwhile, *TpdesatB* rather than *TpdesatA* was clustered closer to the △9 (18 > 16) group in phylogenetic tree. It indicated that C18 served as the substrate of *TpdesatB* prior to C16 in the short‐term cold exposure.

Our results suggest that, during cold exposure at 0°C, *TpdesatA* and *TpdesatB* contributed to the cold tolerance in *T. pui* larvae. *TpdesatA* has contributed to cold hardiness in long term and *TpdesatB* in short term during cold exposure at 0°C. The seasonal expression pattern of *TpdesatA* exhibited a negative correlation with temperature. While *TpdesatB* showed another expression pattern compared to *TpdesatA*, it is contributed to short‐term cold hardiness and positively corresponds to temperature in seasonal expression pattern. These results indicated that *TpdesatA* played a very important role in seasonal cold‐hardening and *TpdesatB* acted as an important regulating substance in short‐term cold exposure and the proportion change of fatty acids in larvae. Different D9D have diverse substrate specificities, *TpdesatA* tends to use C16:0 and C18:0 as substrate, and *TpdesatB* prefers to C18:0.

## Conflict of interest

None declared.
